# Melatonin up-regulates the expression of the GATA-4 transcription factor and increases testosterone secretion from Leydig cells through RORα signaling in an *in vitro* goat spermatogonial stem cell differentiation culture system

**DOI:** 10.18632/oncotarget.22855

**Published:** 2017-12-01

**Authors:** Shou-Long Deng, Yan Zhang, Kun Yu, Xiu-Xia Wang, Su-Ren Chen, De-Ping Han, C. Yan Cheng, Zheng-Xing Lian, Yi-Xun Liu

**Affiliations:** ^1^ State Key Laboratory of Stem Cell and Reproductive Biology, Institute of Zoology, Chinese Academy of Sciences, Beijing 100101, P.R. China; ^2^ Laboratory of Animal Genetics and Breeding, College of Animal Science and Technology, China Agricultural University, Beijing 100193, P.R. China; ^3^ The Mary M. Wohlford Laboratory for Male Contraceptive Research, Center for Biomedical Research, Population Council, New York, NY 10065, USA

**Keywords:** melatonin, RORα, steroid hormone, GATA-4, goat

## Abstract

Because androgen function is regulated by its receptors, androgen-androgen receptor signaling is crucial for regulating spermatogenesis. Androgen is mainly testosterone secreted by testis. Based on the results of early studies in goats, the administration of melatonin over an extended period of time increases steroid production, but the underlying mechanism remains unclear. Here, we report the expression of the melatonin membrane receptors MT1 and MT2 and the retinoic acid receptor-related orphan receptor-alpha (RORα) in the goat testis. An *in vitro* differentiation system using spermatogonial stem cells (SSCs) cultured in the presence of testicular somatic cells was able to support the formation of sperm-like cells with a single flagellum. The addition of 10^-7^ M melatonin to the *in vitro* culture system increased RORα expression and considerably improved the efficiency of haploid cell differentiation, and the addition of the RORα agonist CGP52608 significantly increased the testosterone concentration and expression of GATA binding factor 4 (GATA-4). Furthermore, inhibitors of melatonin membrane receptors and a RORα antagonist (T0901317) also led to a considerable reduction in the efficiency of haploid spermatid formation, which was coupled with the suppression of GATA-4 expression. Based on these results, RORα may play a crucial role in enhancing melatonin-regulated GATA-4 transcription and steroid hormone synthesis in the goat spermatogonial stem cell differentiation culture system.

## INTRODUCTION

Based on earlier findings from our laboratory, melatonin promotes the differentiation of spermatogonia into functional haploid cells in the testes of the Suffolk ram [1]. Our present report extends these findings to the Saanen goat, another short-day breeder. We used an *in vitro* cell culture system that mimics the testes *in vivo* to determine whether retinoic acid receptor-related orphan receptor-alpha (RORα/NR1F1) signaling is also involved in melatonin-promoted goat haploid spermatid production. The interaction of spermatogonial stem cells (SSCs) with the somatic testicular Leydig cells, Sertoli cells and peritubular myoid cells *in vitro* may be particularly important for SSC proliferation and differentiation [2–4]. Mice with a targeted disruption of GATA binding factor 4 (GATA-4) in Sertoli cells display a loss of the establishment and maintenance of the spermatogonial progenitor pool, suggesting that the function of the testicular somatic cells is damaged. Transplantation of germ cells from the testes of early *GATA-4* conditional knockout (cKO) mice or from *in vitro* differentiated SSCs cells to *Kit*^W/W-v^ recipient seminiferous tubules restores spermatogenesis and offspring are obtained [5–7]. *In vitro* culture systems, including the use of organ cultures, seminiferous tubule fragment cultures, and mixed cell co-cultures, have recently been shown to support germ cell differentiation [8–10]. Haploid spermatids with tails have been obtained from these cultures and used to produce normal offspring after round spermatid injection (ROSI), but the differentiation rate was very low [5, 11, 12]. The cell co-culture model provides a similar microenvironment that is analogous to spermatogenesis *in vivo*, which also extends the survival of spermatogenic cells *in vitro* and improves the sperm differentiation rate [13, 14].

Based on increasing evidence, meiosis and sperm maturation are regulated by various hormones, most notably gonadotropin-releasing hormone (LHRH) secreted from the hypothalamus, to influence pituitary gland luteinizing hormone (LH) and follicle stimulating hormone (FSH) release, which regulates testis function [15–18]. As shown in the study by Viguie et al in ewes, *in vivo* administration of melatonin delays the increase in LHRH and LH secretion [19]. According to another *in vivo* study, melatonin administration also increases plasminogen activator activity in ram spermatozoa [20], suggesting that melatonin, a major secretory product of the pineal gland, possesses both lipophilic and hydrophilic properties that allow it to pass through the blood-testis barrier and enter the adluminal compartment [21] where it plays an important role in gametogenesis through a variety of pathways [22, 23]. G protein-coupled receptors are a major signal transduction pathway for melatonin. As a neuroendocrine hormone, melatonin regulates the transcription of animal reproduction genes by binding nuclear receptors [24, 25]. Antioxidant response signaling is another pathway by which melatonin regulates reproductive function [26]. After binding to a membrane-bound receptor, melatonin regulates testosterone synthesis by activing Gi (inhibitory G protein) and its downstream proteins, such as adenylate cyclase (AC) [27]. Through the membrane-associated pathway, melatonin alters gonad and steroid hormone secretion [28]. Melatonin regulates related genes via the RORα pathway [29–31]; for example, melatonin participates in regulating aromatase transcription to promote the conversion of androgen into estrogen [32]. Thus, melatonin may be involved in regulating the intratesticular estrogen level to support spermatogenesis.

In seasonally breeding mammals, melatonin modulates reproductive functions in response to changes in daylight by regulating different levels of the hypothalamic–pituitary–gonadal axis [33]. The melatonin receptor is expressed in testicular cells [34]. By binding to its receptors, melatonin directly influences androgen production by Leydig cells [35], which in turn affects testis development in mice [36]. RORα is a transcriptional regulator of steroid hormone receptor superfamily genes. Through its target genes, RORα exerts important effects on differentiation and development [37]. In the present study, we provide further evidence that RORα increases melatonin-regulated steroid hormone synthesis and SSC differentiation in an *in vitro* Saanen goat SSC/testis somatic cell culture. The pathway by which melatonin regulates steroidogenesis has also been studied. These findings thus provide insights into the treatment of diseases caused by androgen deficiency.

## RESULTS

### RORα expression is up-regulated during development in goat testes

**Figure 1:**
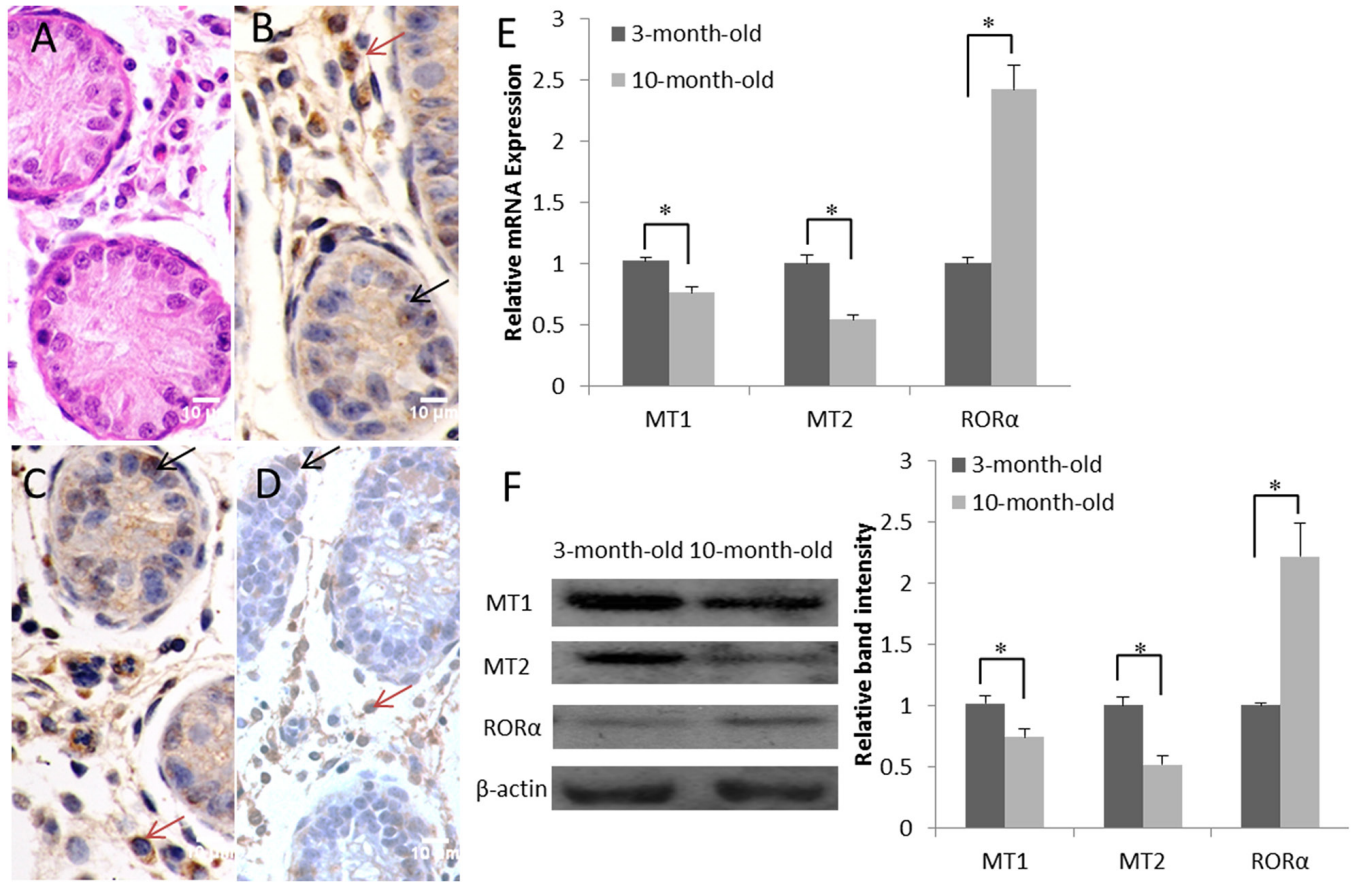
Expression and localization of the melatonin membrane receptors (MT1 and MT2) and nuclear receptor (RORα) in the goat testis **(A)** H&E staining of the 3-month-old goat testis. **(B-D)** Immunohistochemical study of MT1, MT2 and RORα expression in the 3-month-old goat testis; primordial germ cells are indicated by black arrows and Leydig cells are indicated by red arrows. **(E)** Real-time PCR analysis of MT1, MT2 and RORα mRNA levels in the testes at various ages (3 months old and 10 months old). **(F)** Expression of the MT1, MT2, and RORα proteins in the testes at various ages (3 months old and 10 months old) was detected by western blotting. Data are expressed as means ± SEM; ^*^ *P* < 0.05.

In histological sections of the testes, only spermatogonia were detected within the seminiferous tubules of 3-month-old goats (Figure 1A). Immunocytologically, we detected the melatonin receptors MT1, MT2 and RORα in the samples of 3-month-old goat testes. Positive staining resulted in a yellowish or dark brown color. MT1 and MT2 were localized mainly inside the primordial germ cells and were also detected in the Leydig cells (Figure 1B and 1C). RORα was restricted to the perinuclear region of the Leydig cells and was detected at lower levels in Sertoli cells and primordial germ cells (Figure 1D). According to the real-time PCR analysis, both MT1 and MT2 were expressed in the goat testes, and their expression decreased as a function of increasing age. In contrast, RORα mRNA expression increased with aging (Figure 1E). Meanwhile, the MT1, MT2 and RORα contents detected in the western blots were correlated positively with their mRNA levels (Figure 1F).

### Melatonin promotes haploid spermatozoa formation *in vitro*

**Figure 2:**
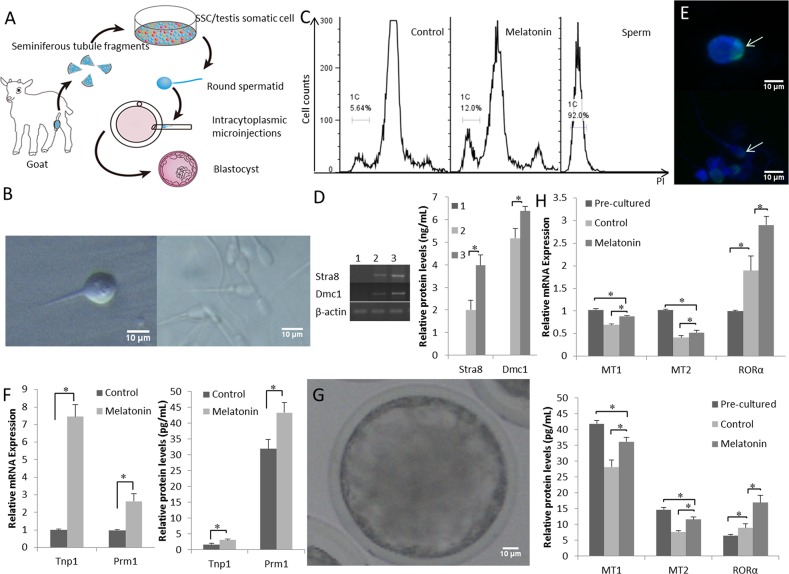
Functional goat haploid spermatozoa were obtained from the *in vitro* testis cell culture **(A)** A schematic illustration of the culture system used in the present study. **(B)** Representative micrographs of a spermatid with a single flagellum isolated from the *in vitro* culture (left) and adult goat sperm used as a control (right). **(C)** DNA contents of the suspended cultured cells were examined by flow cytometry. “Control” is the group that was induced to differentiate with basic medium. “Melatonin” represents cells cultured in basic medium supplemented with melatonin. Adult sperm cells were used as a positive control. 1C marks the haploid peaks. **(D)** The expression of Stra8 and Dmc1 was assessed in suspension cells. 1: cells from the original passage; 2: cells passaged after differentiation; 3: after induction with 10^-7^ M melatonin, suspension cells were passaged after differentiation. **(E)** Haploid cells expressed the mature sperm protein acrosin (green); nuclei of the cells were stained with DAPI (upper panel), and adult goat sperm were used as a control (lower panel). **(F)** Expression patterns of post-meiotic genes (Tnp1 and Prm1). **(G)** Reconstructed embryos developed to the blastocyst stage. **(H)** The expression of the MT1, MT2, and RORα was detected pre-culture and post-culture; differentiated cells and cells cultured without melatonin were used as controls. Data are expressed as means ± SEM. ^*^ *P* < 0.05.

The digested mixed testicular cells were seeded into Petri dishes (Figure 2A). During the early stages of incubation, the somatic cells (Sertoli and Leydig cells) grew in adherent cultures. The goat SSCs were differentiated into developmentally competent sperm-like cells by day 15 of culture (Figure 2B). The late round spermatids (Sa2) or early elongating spermatids (Sb) with a single flagellum and a diameter less than 10 μm were detected, as previously reported [38]. We determined the DNA content of the cells by flow cytometry to investigate whether SSCs differentiate *in vitro*. As shown in Figure 2C, the DNA content of suspended cells showed three peaks, including haploid (1C), prominent diploid, and tetraploid peaks. The haploid efficiency of SSCs treated with additional melatoninwas 13.67 ± 2.31% which was significantly higher than the control group (*P* < 0.05) (Table 1). Groups treated with additional melatoninexpressed higher levels of the SSC differentiation-related genes Stra8 and Dmc1 (Figure 2D).

**Table 1:** Ratio of haploid spermatozoa in suspending cells in 15 days after SSC differentiation

Differentiation medium	Haploid ratio (%)
Control	5.71 ± 1.36 ^b^
Melatonin	13.67 ± 2.31 ^a^

^a, b^ Difference between Melatonin and Control (*P* < 0.05).

Spermatids expressed the mature sperm protein acrosin (Figure 2E). The expression of the post-meiotic genes Tnp1 and Prm1 was up-regulated by melatonin in the differentiated haploid cells compared with that in the control cells (Figure 2F). Single-tailed spermatids were injected into goat metaphase II-stage oocytes and the reconstructed embryos were capable of developing into a blastocyst (Figure 2G). Thus, the *in vitro* cultured spermatids with a single tail had the potential for further development *in vitro*.

The expression of MT mRNAs and protein decreased in the culture system, but MT expression in the melatonin-treated cells was significantly higher than the expression in the control group (*P* < 0.05). The expression of the RORα mRNA and protein was significantly increased in melatonin-treated cells compared with that in the control group (*P* < 0.05) (Figure 2H), suggesting that melatonin promoted goat SSC differentiation in the *in vitro* testis cell culture system.

### Melatonin promotes testosterone secretion through the RORα pathway

**Figure 3:**
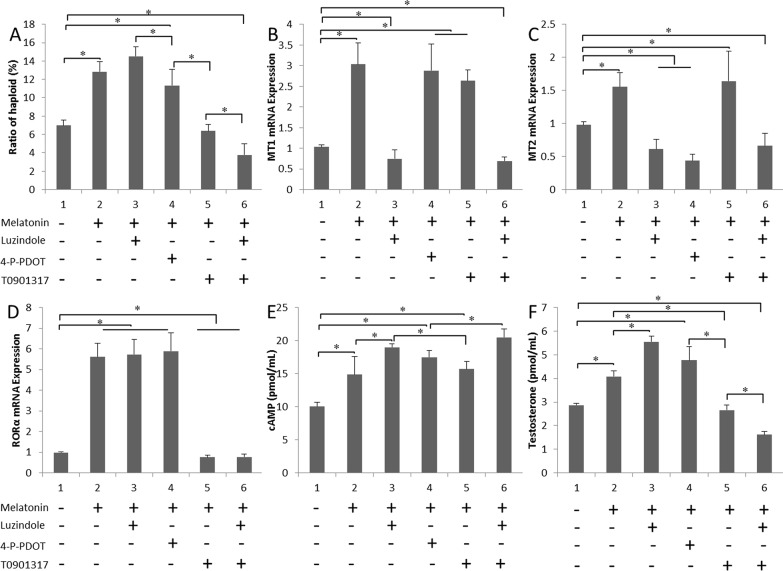
Effects of the melatonin treatment on testosterone secretion **(A)** Ratios of *in vitro* differentiated haploid cells obtained following treatment with luzindole, 4-P-PDOT and T0901317 are shown. **(B-D)** MT1, MT2 and RORα expression in cells cultured with various melatonin receptor antagonists. **(E)** and **(F)** cAMP and testosterone concentrations in cells cultured with various melatonin receptor antagonists. Data are expressed as means ± SEM; ^*^ *P* < 0.05.

Melatonin, luzindole, 4-P-PDOT, and T0901317 were added to the culture system, either alone or in combination. The efficiency of the formation of haploid cells was determined by flow cytometry (Figure 3A). Melatonin receptors were involved in SSC differentiation. The formation of haploid cells was dramatically lower by T0901317 compared with that in the control (*P* < 0.05). The levels of the MT1 and MT2 mRNAs were markedly lower in the melatonin+luzindole group (*P* < 0.05). MT2 expression was lower in the melatonin+4-P-PDOT group (Figure 3B and 3C). MT1 and MT2 expression were not affected in the melatonin+T0901317 group, but RORα expression was lower (*P* < 0.05) (Figure 3D). There was no statistical difference between antagonist group and melatonin+antagonist group (Supplementary Figure 1). The cAMP level was higher in cells treated with additional melatonin than in the untreated cells, which might be due to the long-term melatonin treatment, resulting in enhanced cell sensitivity. Among these six groups, the highest cAMP levels were observed in the luzindole-treated group. No statistically significant difference in cAMP levels was observed between the melatonin+T0901317-treated cells and cells treated with only additional melatonin (Figure 3E). Testosterone secretion was only induced in the groups treated with the melatonin+MT1 inhibitor group (*P* < 0.05). In contrast, steroid production was lower in the T0901317-treated cultures (*P* < 0.05) (Figure 3F).

### Melatonin up-regulates steroidogenesis-related genes via RORα

**Figure 4:**
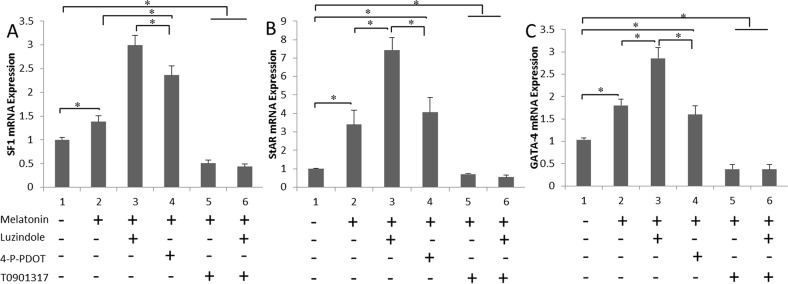
Melatonin up-regulates the expression of steroidogenesis-related genes via RORα **(A and B)** The mRNA levels of the steroidogenesis-related genes SF1 and StAR in cells treated with luzindole, 4-P-PDOT and T0901317 are shown. **(C)** GATA-4 expression patterns in cultured cells treated with the indicated melatonin receptor antagonists are shown. Data are expressed as means ± SEM; ^*^ *P* < 0.05.

Melatonin membrane receptor (MT1 and MT2) antagonists and antagonist of the melatonin nuclear receptor (RORα) were added to the culture media separately. SF1, StAR, and GATA-4 expression in the cultured cells were measured using real-time PCR. Melatonin up-regulated the expression of steroidogenesis-related genes. In contrast, the SF1 and StAR levels were remarkably lower in the T0901317-treated cells (*P* < 0.05) (Figure 4A and 4B). A similar effect of melatonin on GATA-4 mRNA expression was also observed in the testis cell cultures (Figure 4C), suggesting that melatonin up-regulates the expression of steroidogenesis-related genes in the *in vitro* culture via RORα.

### Melatonin promotes GATA-4 expression via altering the RORα signals

**Figure 5:**
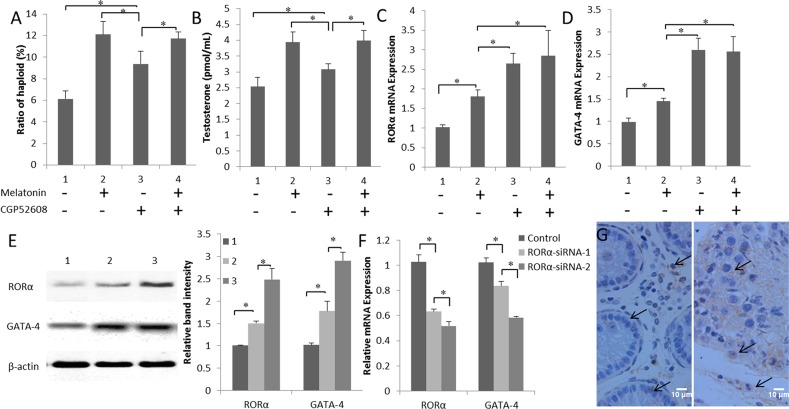
Melatonin promotes GATA-4 expression via the nuclear receptor RORα **(A)** Ratios of *in vitro* differentiated haploid cells in melatonin- and CGP52608-treated cells. **(B)** Testosterone concentrations in cells cultured with CGP52608. **(C)** and **(D)** Expression of the RORα and GATA-4 mRNAs in cells cultured with CGP52608. Data are expressed as means ± SEM; ^*^ *P* < 0.05. **(E)** Expression of the RORα and GATA-4 proteins in cultured cells was detected by western blotting. 1, control; 2, melatonin-treated group; 3, CGP52608-treated group. Data are expressed as means ± SEM; ^*^ *P* < 0.05. **(F)** The expression of the RORα and GATA-4 mRNAs was detected in cells transfected with the RORα siRNA. Data are expressed as means ± SEM; ^*^ *P* < 0.05. **(G)** GATA-4 expression was observed in 3-month-old (left) and 10-month-old (right) goat testes using immunohistochemistry.

Cells in the co-culture system that were treated with the RORα-specific agonist CGP52608 exhibited increases in both the efficiency of the formation of haploid cells and testosterone production compared with those in the control (*P* < 0.05) (Figure 5A and 5B). The highest GATA-4 expression was observed in the CGP52608-treated group compared to the melatonin-treated and control groups (*P* < 0.05) (Figure 5C and 5D). Similar results were obtained in the western blots for the GATA-4 protein levels. Increased RORα expression correlated with the increase in GATA-4 production (Figure 5E). In contrast, interference with RORα expression using two siRNAs significantly decreased the expression of the GATA-4 mRNA (*P* < 0.05) (Figure 5F). In addition, the level of testosterone was decreased in cells transfected with the GATA-4 siRNA (*P* < 0.05) (Supplementary Figure 2). Immunohistochemistry was used to visualize GATA-4 localization in the 3-month- and 10-month-old goat testes. GATA-4 staining was detected in the Leydig cells, Sertoli cells, and primordial germ cells, and increased as a function of goat age (Figure 5G). Based on these results, melatonin promotes steroid synthesis through the nuclear receptor RORα, which may trigger GATA-4 transcription.

## DISCUSSION

Melatonin is known to promote stem cell proliferation and differentiation [39–41]. In addition, melatonin is involved in the function of the male reproductive system, particularly the testes, since Leydig cells are sensitive to melatonin [42, 43]. The simultaneous administration of melatonin with the transplantation of spermatogonial stem cells in azoospermia mouse testes increases the efficiency of transplantation and improves the structural properties of the testis tissue [44]. Here, melatonin membrane receptors and nuclear receptors were detected in goat testes. Moreover, the expression of RORα increased with ageing. In the present study, a mixed culture of dissociated testicular somatic cells with SSCs was used to estimate goat SSC differentiation *in vitro*. After 15 d of co-culture, the differentiation of SSCs into spermatids was detected and sperm cells bearing a single flagellum were observed. The addition of melatonin to this system promoted SSC differentiation into functional haploid sperm-like cells, which was associated with an up-regulation of RORα expression and an induction of the expression of SSC differentiation-related genes, such as Stra8 and Dmc1, consistent with an earlier report [45]. Testosterone concentrations increase in goats receiving subcutaneous injections of melatonin during the non-reproductive season [46]. As shown in the study Rekik et al, a melatonin treatment increases testosterone levels in rams [47]. Increased levels of androgens during winter may be primarily due to the stimulatory effect of melatonin on the steroidogenic enzyme 3beta-hydroxysteroid dehydrogenase [48]. In this study, the addition of exogenous melatonin to the testis cell differentiation-competent culture system also increased testosterone production; furthermore, a RORα-specific agonist increased steroid production.

Melatonin acts through several specific receptors, including membrane receptors (MT1 and MT2) and members of the RZR/ROR nuclear receptor family (RORα) identified in a large variety of mammals [49–51], and exerts a variety of effects that differ between tissues and cells, depending on the receptor [52, 53]. RORα plays a key role in cell differentiation [54]. RORα is significantly down-regulated in T cells stably transfected with an inducible MT1 antisense RNA [55]. Melatonin exhibits potent anti-breast cancer activity mediated via the MT1 receptor. Melatonin also represses the transcriptional activity of some mitogenic nuclear receptors, including RORα, through the MT1 pathway while potentiating the activity of other receptors (RARα and RXRα) involved in differentiation, apoptosis, and inhibiting proliferation [56]. RORα was expressed at much higher levels in the melatonin+luzindole-treated group and was inhibited by MT1/MT2. Melatonin affected both the transcription of RORα and its regulated genes [57, 58]. According to the study by Roth et al, the interaction between melatonin and a nuclear receptor may be responsible for the suppression of PC12 cell growth, because these cells do not express functionally active melatonin receptors on the cell surface [59]. Moreover, melatonin depresses the differentiation of mesenchymal stem cells into fat cells without acting through a membrane receptor [60]. However, melatonin may be transferred into cells after interacting with its nuclear receptor, because it is a lipid soluble hormone [61]. Luzindole is blocks the binding of melatonin to MT1/MT2. Melatonin treatment desensitizes MT1 receptors, whereas luzindole increases ligand binding and G-protein activation. Luzindole also stimulates downregulation of the MT1 receptor protein, interfering with the synthesis and/or degradation of the receptor [62]. In the present study, the control group contained endogenous melatonin which may play a role during *in vitro* culture without the addition of melatonin, lead to the expression of MT1/MT2 is higher than that of luzindole treatment group. Melatonin regulated the expression of the steroid hormone-related gene StAR through a nuclear receptor, suggesting that melatonin penetrated the testes and regulated steroidogenesis via various signaling pathways.

The classic function of melatonin is to decrease the cAMP concentration through G-proteins and subsequently down-regulate CREB [63]. Acute melatonin treatment of INS-1 cells inhibits cAMP-mediated signal transduction. However, prolonged exposure of INS-1 cells to melatonin sensitizes cAMP-mediated responses to forskolin [64]. Mimicking the short photoperiod, melatonin signaling (16 h exposure) in primary cultures of melatonin target cells obtained from the ovine pars tuberalis increases the cAMP response to forskolin stimulation compared to that in untreated cells, a phenomenon termed sensitization [65]. In our study, cAMP levels were increased during long-term melatonin treatment. When the melatonin receptor was blocked, the cAMP concentration increased, possibly due to melatonin receptor sensitivity and antioxidant activity. The expression levels of RZR/RORs are regulated by cAMP [66]. RORα activates CYP8B1 promoter reporter activity in cAMP-stimulated human and mouse cells [67]. The cAMP response unit also contains a putative response element for RORα [68]. In our studies, cAMP and RORα were up-regulated in melatonin+luzindole group, and we further characterized the effect of the RORα response on cAMP-induced transcription.

CREB phosphorylation and its binding to cAMP response elements are two crucial steps in the cAMP pathway. The binding of phosphorylated CREB to the cAMP response element of the StAR promoter accelerates steroid synthesis. However, in steroidogenic cells, not all cAMP-regulated genes, such as StAR, have a consensus cAMP response element (CRE), but many of them have a regulatory sequence recognized by a GATA family transcription factor [69]. StAR transcription is activated by two transcription factors, SF1 and GATA-4 [70]. In the testes, GATA-4 predominantly regulates StAR transcription [71]. GATA-4 is required for the expression of some steroidogenic genes, including SF1 and StAR [72]. Increased levels of melatonin are known to suppress testis function [73]. Some studies have shown that the inhibitory effects of melatonin on testosterone production are mediated by the down-regulation of GATA-4 and SF1 expression in a mouse MA-10 Leydig cell line [74]. But melatonin improves testis function in short-day seasonal breeders [75]. The expression level of the melatonin receptor in the goat thymus increases considerably in winter [76]. In addition, melatonin increases both the blood testosterone concentration and acrosin activity in ram spermatozoa [77]. Based on the results of the present study, melatonin promoted testosterone production and RORα-enhanced GATA-4 and SF1 expression. This result is further supported by the high degree of homology between RORα and RXR (retinoid X receptor) [78]. Retinoid-responsive StAR transcription is largely regulated by an RXR/RAR heterodimeric motif in the mouse StAR promoter [79]. Moreover, retinoid receptors are capable of increasing the expression of the StAR protein [80], and RXRα interacts directly with GATA-4 [81]. In the present study, both RORαandGATA-4 were expressed in the goat testis in an age-dependent manner.

**Figure 6:**
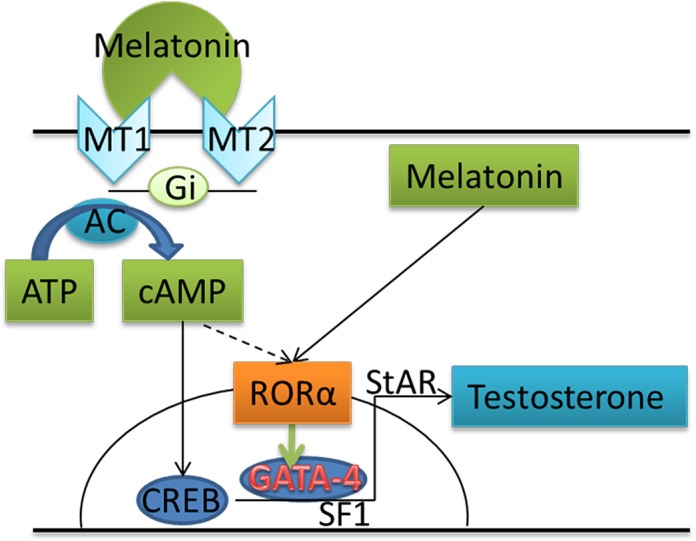
Schematic illustrating the proposed mechanism by which melatonin increases testosterone production in goat testis cells by binding to RORα

In summary, we confirmed that both melatonin membrane receptors and the nuclear receptor are expressed in the goat testes. When spermatogenic cells in the *in vitro* culture system are treated with melatonin, the expression of GATA-4 and its downstream genes was up-regulated and testosterone synthesis was promoted, likely enhancing the efficiency of producing haploid cells from SSCs. Through RORα, melatonin promotes GATA-4 expression to stimulate testosterone synthesis by Leydig cells, which in turn supports more efficient meiosis and spermatid differentiation via spermiogenesis. Meanwhile, cAMP promotes RORα expression (Figure 6).

## MATERIALS AND METHODS

### Chemicals and reagents

All chemicals used in this study were purchased from Sigma-Aldrich Chemical Company (St. Louis, MO, USA) unless stated otherwise.

### Ethics statement

Goat surgical biopsies were performed at the experimental station of the China Agricultural University, and the whole procedure was conducted in accordance with the protocol approved by the Animal Welfare Committee of China Agricultural University (Protocol Number XK662).

### Isolation of male goat testicular cells

Testis tissues were obtained from three 3-month-old Saanen goats. Some of the tissues were fixed with 4% paraformaldehyde, embedded in paraffin wax, sectioned, and stained with hematoxylin and eosin (H&E) for the histological analysis. Other testis tissues were transported to the laboratory in normal saline and washed thrice with phosphate-buffered saline (PBS) supplemented with 100 IU/mL penicillin and 100 mg/mL streptomycin. After decapsulation, the dissociated cells and the short seminiferous tubule fragments were dissociated using a modified enzymatic digestion procedure [82]. Briefly, the fragments were incubated with a 10-fold dilution of an (w/v) enzyme cocktail containing 1 mg/mL collagenase type IV, 1 mg/mL hyaluronidase, and 5 μg/mL DNase I at 37°C for 15 min, followed by neutralization with Dulbecco's Modified Eagle's Medium (DMEM) containing 10% fetal bovine serum (FBS) (Gibco, Grand Island, NY, USA). The cell suspension was filtered using a 40 mesh sieve. The dispersed cells were washed twice with DMEM and pelleted by centrifugation at 500 *g*.

### *In vitro* differentiation of SSCs

Autologous suspensions of somatic testicular cells (containing spermatogonial stem cells, Sertoli cells, peritubular myoid cells and Leydig cells) were prepared using a method reported by Zhang et al [83], with minor modifications. Briefly, cells were seeded in 60 mm culture dishes and incubated at 37.8°C for 3 d. The temperature was then maintained at 34°C in a humidified atmosphere of 5% CO_2_/air (v/v). Cells were suspended in DMEM supplemented with 5% FBS, 1% non-essential amino acids, 10 ng/mL stem cell factor, 10 ng/mL fibroblast growth factor, 25 ng/mL epidermal growth factor, 10 ng/mL insulin-like growth factor, 20 ng/mL glial-derived neurotrophic factor, 10 μg/mL transferrin, 10^−4^ M vitamin C (Vc), 4 mM L-glutamine, 0.05 IU/mL FSH, 0.05 IU/mL LH and 1% penicillin–streptomycin [84]. Then, 10^-7^ M melatonin was added to the cultures. In a subset of cultures, melatonin (10^-7^ M) was added with either luzindole (10^-7^ M) (a non-selective MT1/MT2 inhibitor), 10^-7^ M 4-P-PDOT (a MT2-specific inhibitor), 10^-6^ M T0901317 (a RORα antagonist) or 10^-7^ M CGP52608 (a RORα-specific agonist) [85]. The other tested activators and/or inhibitors were added to the culture medium. In the culture system, half of the media were changed every 2 d. The rate of cell growth was monitored. Cells were cultured for 15 d and transfected with a RORα-siRNA (target sequence: 1 GAGCCTTATGTGGACGGCA and 2 AGCGATGAAAGCTCAAATTGAAA) and GATA4-siRNA (target sequence: AACCTTAACAAATCA AAGACACC) using the INTERFERin siRNA transfection reagent (Polyplus-transfection, Illkirch, France) according to the manufacturer's instructions.

### Flow cytometric analyses

The DNA content of the cells was examined by flow cytometry. The *in vitro* cell suspensions were adjusted to 1×10^6^ cells/mL and collected at 15 d; sperm from a mature goat served as a control. The cells and sperm were fixed with 70% ethanol for 4 h. After three washes with PBS, the cells were incubated with PBS containing 200 μg/mL RNase I and 20 μg/mL propidium iodide (PI) at 37°C for 10 min. Finally, the DNA content of the cells was determined by flow cytometry.

### Quantitative real-time PCR and reverse transcription PCR

The testis tissues and testicular cells were collected to determine the expression levels of specific genes, including melatonin receptors (MT1, MT2 and RORα), genes expressed in post-meiotic spermatids and sperm (transition protein 1 (Tnp1) and protamine 1 (Prm1)), rate-limiting enzymes in testosterone synthesis (steroidogenic acute regulatory protein (StAR) and splicing factor 1 (SF1)) and GATA-4. Genes related to SSC differentiation, stimulated by retinoic acid gene 8 (Stra8) and DNA meiotic recombinase 1 (Dmc1), were detected by reverse transcription PCR, and *β*-actin was used as an internal control. The primer sequences are listed in Table 2. Total RNA was extracted using TRIzol reagent (Invitrogen, Carlsbad, CA, USA) according to the manufacturer's protocol. Reverse transcription PCR was performed using a cDNA synthesis kit (Promega, Madison, WI, USA), and 2 μL of total RNA were used according to the manufacturer's protocol. Real-time PCR reactions were performed using a Real Master Mix SYBR Green Kit (Tiangen, Corp., Beijing, China) on a Stratagene Mx300p (Agilent Technologies Inc., Santa Clara, CA, USA). Fold changes in gene expression were calculated using the *2^-ΔΔct^* method as a ratio of the expression levels of the treated groups to the expression level of the control group.

**Table 2:** The primer sequences

Gene (Accession NO.)	Primer sequence	Product size (bp)
MT1(AB716764.1)	5’ GCGTCATCGGGTCTGTTTTC 3’	175
	5’ AGGGTCCCCACACACAGGT 3’	
MT2(JF266705.1)	5’ GCGTCTACTCGTGCCCCTT 3’	159
	5’ TGCTCTCCGCCTTGACCTT 3’	
RORα(NM001285652.1)	5’ CTTCACGATGACCTCAGCAACTA 3’	182
	5’ TAGGGGAAGAAGCCTGATGC 3’	
Stra8(JQ836663.1)	5’ CCTTGGAGCGGACACAGAA 3’	175
	5’ CTTTTGTCCAGGAAACTTGCC 3’	
Dmc1(KR935229.1)	5’ GGTGGCTACTCAGGAGGAAAGA 3’	187
	5’ GGAACTTCGCTGCTACATAATCA 3’	
SF1(XM013967971.1)	5’ TACCTCTACCCTGCCTTCCCT 3’	177
	5’ CCGCACTTGGTCCTCATCA 3’	
StAR(XM013975437.1)	5’ GCAGAAGGGTGTCATCAGAGC 3’	172
	5’ GGCAAAATCCACTTGGGTCT 3’	
GATA-4(XM013965849.1)	5’ ACCAGAAAACGGAAGCCCA 3’	156
	5’ GGGCTCTGTTTTGATGGGAC 3’	
Tnp1(XM005676523.2)	5’ TGAGGAGGGGCAAGAACAGA 3’	134
	5’ TCACAAGTGGGAGCGGAAAT 3’	
Prm1(HM773246.1)	5’ AAGATGTCGCAGACGAAGGAG 3’	112
	5’ GGTCTTGCTACTGTGCGGTTA 3’	
*β*-actin(AF481159.1)	5’ CACGGTGCCCATCTACGAG 3’	158
	5’ CCTTGATGTCACGGACGATTT 3’	

### Enzyme-linked immunosorbent assay

The cultured cells and spent media were collected. Enzyme-linked immunosorbent assay (ELISA) kits were used to detect the Stra8, Dmc1, Prm1, Tnp1, MT1, MT2, RORα, testosterone and cAMP levels (Hermes Criterion Biotechnology, Vancouver, Canada) according to the manufacturer's instructions.

### Immunofluorescence analysis

The cells in the culture system were processed for immunofluorescence staining for acrosin (Bioss, Beijing China, bs-5151R), a marker of differentiated spermatozoa, after 15 d. Briefly, the cells were fixed with 70% alcohol for 2 h and then washed twice with PBS. Slides were blocked with 1% BSA for 1 h at room temperature, and the antibody (final concentration 1:200) was added to the solution and incubated for 4 h. Slides were rinsed twice and washed three times with PBS for 5 min. The secondary antibody (1:500) was incubated with the cells for 1 h at room temperature, followed by washes with PBS. The nuclei were stained with DAPI.

### Immunohistochemistry

Testis samples from 3-month-old goats were fixed with 4% paraformaldehyde. The samples were cryo-embedded in optimum cutting temperature (OCT) compound and then cut into 7 μm thick sections. MT1 (Santa Cruz Biotechnology sc-13180), MT2 (Santa Cruz Biotechnology sc-13177), RORα (Abcam ab60134) and GATA-4 (Santa Cruz Biotechnology sc-1237) distributions were examined using immunohistochemistry. Briefly, after washing three times with PBS, the slides were incubated with PBS containing 1% bovine serum albumin (BSA) for 1 h at room temperature. Primary antibodies directed against MT1 (final concentration 1:200), MT2 (final concentration 1:200), GATA-4 (final concentration 1:200), and RORα (final concentration 1:200) were added to the solution. After 4 hours of incubation, the secondary antibody was applied for 1 h. Staining was visualized using a 3,3’-diaminobenzidine (DAB) substrate kit.

### Western blot analysis

Total protein was isolated from both cultured cells and goat testes. MT1, MT2, RORα, and GATA-4 were examined by western blotting, and *β*-actin served as a control. The proteins were electrophoretically separated on 12% SDS-PAGE gels under reducing conditions and transferred to nitrocellulose membranes. The blots were blocked in 5% BSA and incubated with the primary antibody overnight at 4°C, followed by incubation with the secondary antibody for 1 h at room temperature. Protein bands were visualized using enhanced chemiluminescence detection reagents (Applygen Technologies Inc., Beijing, China). Optical densities were quantified by scanning densitometry and expressed in arbitrary units determined by ImageJ software (NIH, USA).

### Intracytoplasmic microinjections

Ovaries were obtained from a slaughterhouse. Cumulus cells and cumulus-oocyte complexes (COCs) were selected and cultured in the *in vitro* maturation (IVM) medium (10% FBS TCM-199 (Gibco), 0.05 IU/mL FSH, 0.05 IU/mL LH, 1 μg/mL 17β-estradiol, 10 ng/mL epidermal growth factor, and 2 mM glutamine). Oocytes extruding the first polar body were selected for haploid cell injection. Spermatids with single flagella used for microinjections were less than 10 μm in diameter. Micromanipulation was performed in TCM-199 supplemented with 5 μg/mL cytochalasin B, 3 mg/mL BSA, and 0.5 mM HEPES. Oocytes were activated with 5 μM ionomycin for 5 min prior to the injection. The cell membranes of spermatids with a single flagellum were destroyed by repeated blowing with an injection needle, and then injected into the cytoplasm of oocytes. The intracytoplasmic injection was completed within 1 hour after activation. Recovered couplets were transferred into development medium (*in vitro* culture, the medium included modified synthetic oviduct fluid supplemented with amino acids, 0.2 mM glutamine, 6 mg/mL BSA, 3% essential amino acids, 1% non-essential amino acids and 0.5 mg/mL inositol) for recovery at 38°C in a 5% CO_2_/95% air (v/v) atmosphere for 30 min, and subsequently activated with 5 μM ionomycin for 5 min and 2 mM 6-dimethylaminopyridine for 4 h. After activation, reconstructed embryos were cultured at 38°C in a 5% CO_2_ atmosphere to allow development, and blastocysts were observed on day 7.

### Statistical analyses

All experiments were repeated at least 3 times, the sample size (n = 3) in each experiment. One-way ANOVA followed by Duncan's test was used to determine the statistical significance of the differences between the selected groups. Statistical analyses were conducted using Statistical Analysis System software (SAS Institute, Cary, NC, USA). All data are expressed as means ± standard errors of the means (SEM). Differences were considered significant when *P* < 0.05.

## SUPPLEMENTARY MATERIALS FIGURES


