# Supplementary material for: Melatonin up-regulates the expression of the GATA-4 transcription factor and increases testosterone secretion from Leydig cells through RORα signaling in an in vitro goat spermatogonial stem cell differentiation culture system
Source: Oncotarget. 2017 Dec 1;8(66):110592–605. doi: 10.18632/oncotarget.22855 (PMC5746406; doi:10.18632/oncotarget.22855)
Supplement: Supplementary file 1 [file oncotarget-08-110592-s001.pdf]

Melatonin up-regulates the expression of the GATA-4 transcription factor and increases testosterone secretion from Leydig cells through RORα signaling in an *in vitro* goat spermatogonial stem cell differentiation culture system

SUPPLEMENTARY MATERIALS

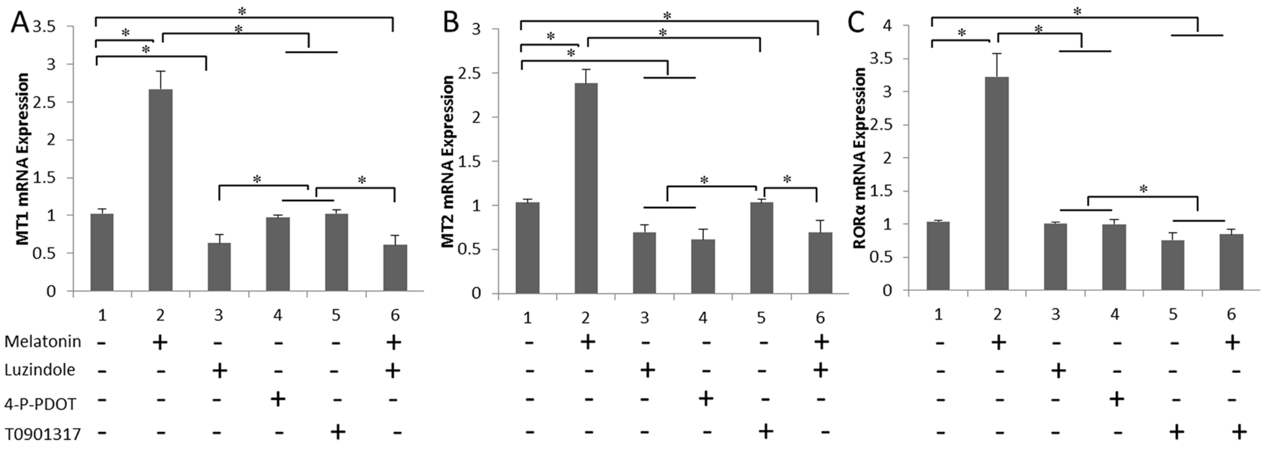

Supplementary Figure 1: (A-C) MT1, MT2 and RORα expression in cells cultured with various melatonin receptor antagonists. Data are expressed as means ± SEM; * $P < 0.05$.

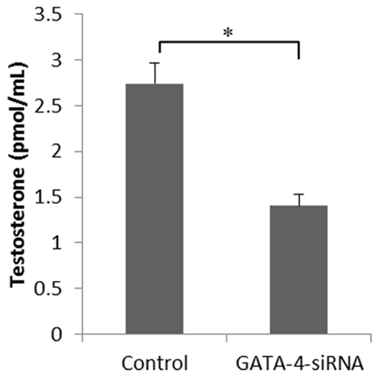

Supplementary Figure 2: The level of testosterone was detected in cells transfected with the GATA-4 siRNA. Data are expressed as means ± SEM; * $P < 0.05$.
